# Real-Time Continuous Glucose Monitoring During the Coronavirus Disease 2019 Pandemic and Its Impact on Time in Range

**DOI:** 10.1089/dia.2020.0649

**Published:** 2021-03-02

**Authors:** Joost van der Linden, John B. Welsh, Irl B. Hirsch, Satish K. Garg

**Affiliations:** ^1^ Dexcom, Inc., San Diego, California, USA.; ^2^ Department of Medicine, University of Washington, Seattle, Washington, USA.; ^3^ Barbara Davis Center for Diabetes, University of Colorado Anschutz Medical Campus, Aurora, Colorado, USA.

**Keywords:** Continuous glucose monitoring, COVID-19, Time-in-range (TIR), Socioeconomics

## Abstract

***Background:*** The coronavirus disease 2019 (COVID-19) pandemic disrupted the lives of people with diabetes. Use of real-time continuous glucose monitoring (rtCGM) helped manage diabetes effectively. Some of these disruptions may be reflected in population-scale changes to metrics of glycemic control, such as time-in-range (TIR).

***Methods:*** We examined data from 65,067 U.S.-based users of the G6 rtCGM System (Dexcom, Inc., San Diego, CA) who had uploaded data before and during the COVID-19 pandemic. Users associated with three counties that included the cities of Los Angeles, Chicago, and New York or with five regions designated by the Centers for Disease Control and Prevention (CDC) were compared. Public data were used to associate regions with prepandemic and intrapandemic glycemic parameters, COVID-19 mortality, and median household income.

***Results:*** Compared with an 8-week prepandemic interval before stay-at-home orders (January 6, 2020, to March 1, 2020), overall mean (standard deviation) TIR improved from 59.0 (20.1)% to 61.0 (20.4)% during the early pandemic period (April 20, 2020 to June 14, 2020, *P* < 0.001). TIR improvements were noted in all three counties and in all five CDC-designated regions. Higher COVID-19 mortality was associated with higher proportions of individuals experiencing TIR improvements of ≥5 percentage points. Users in economically wealthier zip codes had higher pre- and intrapandemic TIR values and greater relative improvements in TIR. TIR and pandemic-related improvements in TIR varied across CDC-designated regions.

***Conclusions:*** Population-level rtCGM data may be used to monitor changes in glycemic control with temporal and geographic specificity. The COVID-19 pandemic is associated with improvements in TIR, which were not evenly distributed across the United States.

## Introduction

Real-time continuous glucose monitoring (rtCGM) data are used to guide individual diabetes management decisions, but their utility for monitoring the adequacy of glycemic control in specific geographic regions or in response to global health crises is inadequately studied. Severe acute respiratory syndrome coronavirus 2 (SARS-CoV-2) or coronavirus disease 2019 (COVID-19) is disrupting economic, social, and personal behaviors worldwide. People with chronic metabolic diseases such as diabetes are disproportionately affected for higher morbidity and mortality from the COVID-19 pandemic.^1^ Earlier studies from Italy^2–5^ have used CGM and flash glucose monitoring data to associate the pandemic with variable improvements in time spent in the 70–180 mg/dL target range (TIR). Similar findings have been reported for CGM users in Spain^6^ and England.^7^ An Israeli study noted associations between pandemic-related changes in TIR and socioeconomic status.^8^ Here we describe efforts to assess temporal changes in glycemic control among rtCGM users in specific areas of the United States affected by the COVID-19 pandemic and relate them to region-specific median household income.

## Methods

We examined data from users of the G6 CGM System (Dexcom, Inc., San Diego, CA) with postal codes in the United States who had started using the system on or before January 1, 2020; had used the mobile app (as opposed to the dedicated receiver) to view their data; had uploaded at least one sensor glucose value each month of the first half of 2020; and had uploaded ≥200 sensor glucose values per day for ≥4 days per week in the prepandemic and intrapandemic observation windows (defined as the 8 weeks ended March 1, 2020, and June 14, 2020, respectively). The requirements for ≥200 daily sensor glucose values for ≥4 days per week were relaxed for an analysis of nationwide TIR values for the 8-week interval ended August 9, 2020. Postal codes allowed assignment of each user to a state and county of residence. County-level COVID-19 prevalence and mortality data as of May 21, 2020, were aggregated by *The New York Times*^9^ and obtained from GitHub.^10^ Timing of stay-at-home orders was reported by the Kaiser Family Foundation.^11^ Detailed analysis was undertaken for Los Angeles County, California (LA), Cook County, Illinois (Cook), and the five counties comprising New York City, New York (NYC) because of their high COVID-19 burdens. Summary statistics were also calculated for each of the five Centers for Disease Control and Prevention “Integrated Food Safety Centers of Excellence” headquartered in Colorado (10 states), Minnesota (10 states), New York (12 states and the District of Columbia), Tennessee (11 states, Puerto Rico, and the U.S. Virgin Islands), and Washington (7 states and Guam).^12^ Each patient's tenure with the G6 System was the interval between their first and most recent uploaded G6 data.

Consensus definitions and goals^13^ were used that included TIR, hypoglycemia-Time Below Range, hyperglycemia- Time Above Range, and clinically meaningful differences. The glucose management indicator (GMI) is a linear function of mean glucose values as previously described.^14^ The dependent *t*-test for paired samples was used for prepandemic and intrapandemic TIR comparisons; the simple linear Pearson correlation coefficient was used to test the relationship between regional disease burden in counties with ≥200 users and the prevalence of patients with five-point increases in TIR. The analysis was consistent with Dexcom's privacy policies^15^ and all patient-specific identifiers were removed before data analysis was begun. It was not registered as a trial.

## Results

### Overall population-level results

**FIG. 1.**
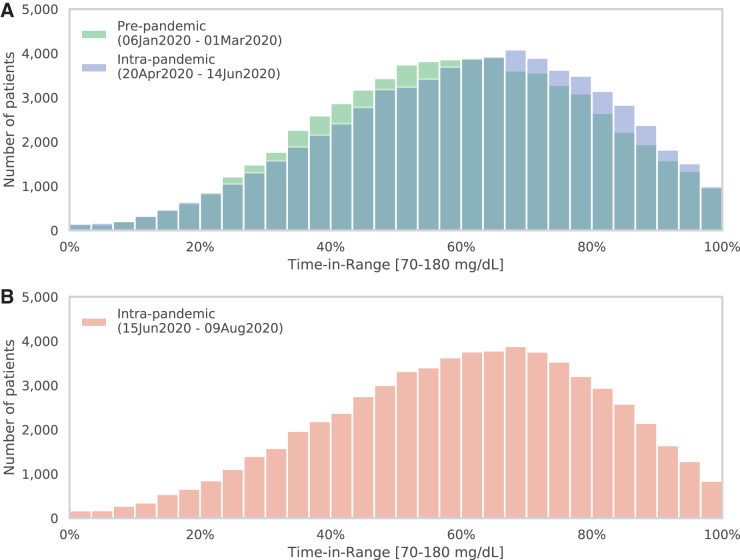
Distribution of 65,067 individuals according to TIR values during prepandemic (green) and intrapandemic (blue) intervals **(A)**. **(B)**, distribution of TIR values from a more recent intrapandemic interval. TIR, time in range.

Comparing data from 65,067 individual uploaders in prepandemic versus early intrapandemic intervals, the mean (standard deviation [SD]) TIR improved by 2.0% (10.0), from 59.0% (20.1) to 61.0% (20.4), *P* < 0.001. An improved TIR was observed in 59.4% of individuals. The population's mean (SD) glucose value decreased from 173.3 (35.9) to 170.2 (36.5) mg/dL (*P* < 0.001), equivalent to a decrease in GMI from 7.46 (0.86)% to 7.38 (0.87)% (*P* < 0.001). The proportion of individuals meeting the 70% or higher TIR goal increased from 31.7% to 36.4% (*P* < 0.001). Figure 1A shows the distribution of prepandemic and intrapandemic TIR values with a favorable (rightward) shift toward higher TIR values. A total of 21,423 (32.9%) individuals experienced clinically meaningful increases in TIR of at least 5 percentage points, compared with 12,121 (18.6%) who experienced decreases of at least 5 percentage points. Figure 1B shows TIR distribution for a more recent intrapandemic interval of June 15 to August 9, 2020, in which the rightward shift was maintained.

The correlation between the logarithm of the number of deaths as of May 21, 2020, and the fraction of patients who experienced an improvement in TIR of at least 5 percentage points in the 100 counties with ≥200 users was also examined. The correlation (*r* = 0.40) was statistically significant (*P* < 0.001); counties with more deaths tended to have more patients with clinically meaningful improvements in TIR.

### Trajectories for Cook, LA, and NYC

**FIG. 2.**
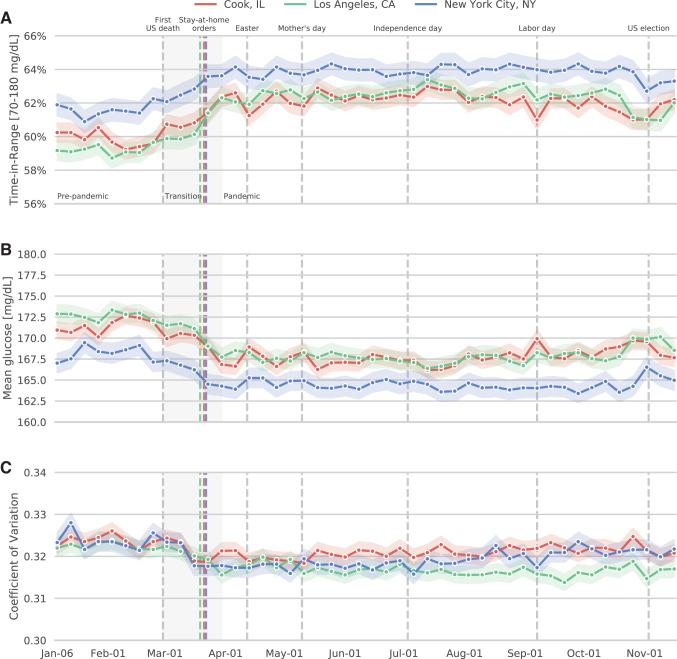
Mean (±SEM) proportions of TIR, glucose values, and glycemic variability in early 2020. Red, Cook County, Illinois; green, Los Angeles County, California; blue, New York City, New York. Vertical lines indicate the first U.S. death attributed to coronavirus disease 2019 (initially given as February 29), issuance of region-specific stay-at-home orders (Cook: March 21; New York: March 22; Los Angeles: March 19), Easter (April 12), Mother's Day (May 10), Independence Day (July 4), Labor Day (September 7), and the U.S. election (November 3). The shaded transition period extends from February 29 to March 30. **(A)** TIR; **(B)** Sensor glucose; **(C)** Coefficient of variation. SEM, standard error of the mean.

Trajectories for TIR, mean glucose, and coefficient of variation in LA, Cook, and NYC near the onset of the pandemic are shown in Figure 2. In all regions, TIR improved after issuance of stay-at-home orders, and the extent of regional differences decreased (Fig. 2A). Each of the TIR improvements between prepandemic and intrapandemic intervals were statistically significant (all *P* < 0.001). These changes were accompanied by decreases in mean glucose value (Fig. 2B) and smaller changes in glycemic variability (Fig. 2C). No similar improvement in TIR was seen in the early months of 2019 (not shown).

**FIG. 3.**
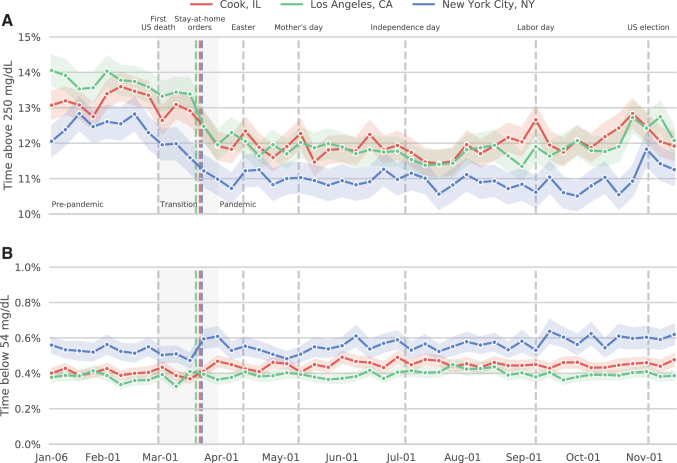
Mean (±SEM) percentages of uploaded continuous glucose monitoring values that were >250 mg/dL **(A)** or <54 mg/dL **(B)**.

The improvements in TIR were largely due to changes in hyperglycemia evidenced by the proportion of glucose values >250 mg/dL (Fig. 3A), rather than hypoglycemia evidenced by the proportion of glucose values <54 mg/dL (Fig. 3B). Before the pandemic, the goal of having <5% of glucose values in the >250 mg/dL range was met by 32.8% of patients; this proportion increased to 37.7% during the pandemic.

### Income and regional disparities

**FIG. 4.**
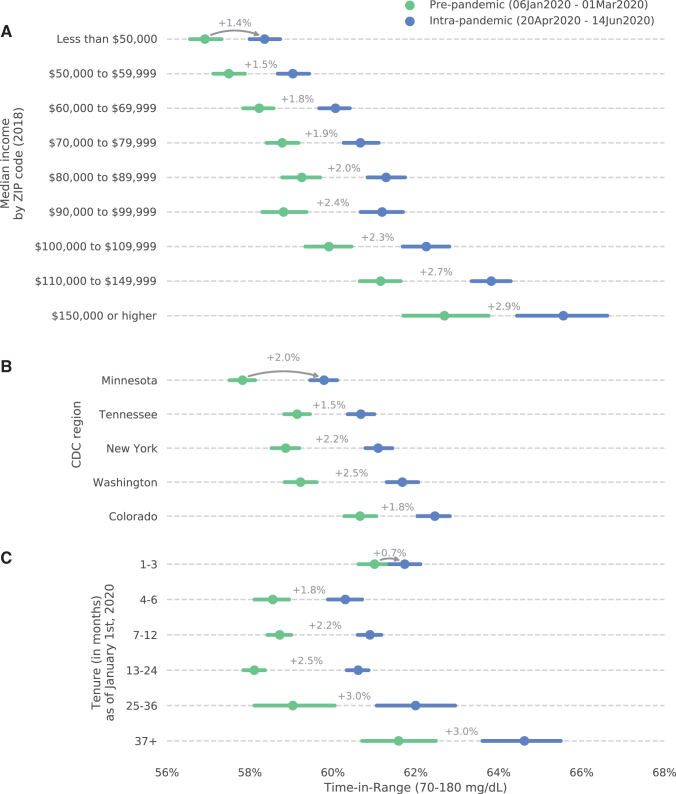
Relationship between prepandemic (green) and intrapandemic (blue) mean TIR values for patients in ZIP codes with different median household incomes **(A)**, living in different CDC-designated “Integrated Food Safety Centers of Excellence” regions **(B)**, and having different tenures with the G6 System **(C)**. Error bars represent 95% confidence intervals. CDC, Centers for Disease Control and Prevention.

As shown in Figure 4, improvements in TIR were not evenly distributed. Mean TIR values were generally higher for patients in zip codes with higher median income, both before and during the pandemic, and pandemic-related improvements in TIR were highest in areas with median household incomes in excess of $150,000 per year (Fig. 4A). Patients in the 10-state “Integrated Food Safety Centers of Excellence” region headquartered in Denver, Colorado, had the highest mean TIR values, both before and during the pandemic, and the largest improvement was seen for patients in the 7-state region headquartered in Seattle, Washington (Fig. 4B). Patients with the most experience with the G6 System (tenures of ≥37 months) had higher TIR values before and during the pandemic than patients who began using the system more recently (Fig. 4C).

## Conclusions

The COVID-19 pandemic emphasizes the need for good glycemic control in patients with diabetes, in large part because most observational studies have reported poorly controlled diabetes is associated with higher risk for hospitalization and death from the viral illness.^16,17^ Conversely, COVID-19 complicates management and may contribute to the pathogenesis of diabetes and its complications.^18^ The impact of the early stages of the pandemic on glycemic control in several case reports has been discussed,^2–8^ as has the role of nonprofit organizations and virtual specialty clinics.^19,20^ The pandemic is also speeding the transitions to virtual diabetes management and adoption of telehealth.^21,22^ This study adds to the existing literature on the adequacy of glycemic control in the COVID-19 pandemic and is among the first to report on outcomes in multiple geographic regions within the United States.

The time-dependent changes shown for three selected counties are likely due to a combination of factors such as pandemic-related reductions in meals eaten outside the home, a more regular stay-at-home lifestyle, and/or increased vigilance toward diabetes management. The observed correlation between higher COVID-19 burden and higher proportions of individuals with meaningfully improved TIR may reflect a relationship between proximity to COVID-19 outbreaks and increased attention to good glycemic control.

Elsewhere in this Supplement, Garg and Norman discuss economic issues related to the COVID-19 pandemic for people with diabetes.^23^ As shown here, the favorable TIR effects of the COVID-19 pandemic accrued disproportionately to patients in areas with relatively high median household income and to early adopters/long-term users of the G6 System. Social inequalities in diabetes care^24^ may be related to the diffusion of innovative diabetes technologies such as rtCGM.^25^

Strengths of the study include the large number of observed CGM users, our reliance on a single CGM system, good estimates of individuals' TIR because of the high data density, and the geographic specificity provided by postal codes. Although we had no control group, we were able to assess glycemic control in areas with widely differing COVID-19 burdens. Limitations of the study include our inability to quantify the populations' age distributions, diagnoses, racial and ethnic backgrounds, type of diabetes and comorbidities, medication regimens, or adoption of other diabetes-related technologies. We are also unable to characterize individual behavioral changes or circumstances. The overall pandemic-related differences in TIR are statistically significant and may be of clinical significance.

Our results suggest a potential role for rtCGM in monitoring acute changes to the health status of populations during a change of public health. In this way, CGM systems could join other wearable devices used to predict and monitor disease outbreaks^26^ or monitor risk factors for the development of diabetes.^27^ CGM systems will continue to provide actionable insights for individuals with diabetes and may also inform discussions of economic and regional health disparities.
